# The ratio of monocytes to lymphocytes multiplying platelet predicts incidence of pulmonary infection-related acute kidney injury

**DOI:** 10.1186/s40001-022-00906-6

**Published:** 2022-12-27

**Authors:** Bo Shen, Zhouping Zou, Yang Li, Ping Jia, Yeqing Xie, Shaomin Gong, Jie Teng, Jiarui Xu, Cheng Yang, Xiaoqiang Ding

**Affiliations:** 1 Department of Nephrology, Zhongshan Hospital, Fudan University, Shanghai, China; 2 Shanghai Medical Center of Kidney, Shanghai, China; 3 Shanghai Key Laboratory of Kidney and Blood Purification, Shanghai, China; 4 Department of Nephrology, Xiamen Branch, Zhongshan Hospital, Fudan University, xiamen, Fujian, China; 5 Nephrology Clinical Quality Control Center of Xiamen, Xiamen, Fujian China; 6 Department of Urology, Zhongshan Hospital, Fudan University, Shanghai, China; 7 Shanghai Key Laboratory of Organ Transplantation, Shanghai, China; 8 Zhangjiang Institute of Fudan University, Shanghai, China

**Keywords:** Acute kidney injury, Pulmonary infection, Monocyte, Macrophages, Risk factor

## Abstract

**Background:**

Inflammation is a crucial factor in the pathogenesis and development of acute kidney injury (AKI). Macrophages, as an important innate immune cell, regulate immune response and play a pathophysiological role in AKI. This study aimed to evaluate the predictive capacity of peripheral blood monocytes for the incidence of pulmonary infection-related AKI.

**Methods:**

We recruited 1038 hospitalized patients with pulmonary infections from January 1 to December 31, 2019, in Zhongshan Hospital, Fudan University. Patients were divided into derivation and validation cohorts. Data on demographic characteristics, disease history, and biochemical indexes were retrieved from the electronic medical system. The composite inflammatory indexes were calculated as monocyte/(lymphocyte × platelet ratio) (MLPR). We applied dose–response relationship analyses to delineate the nonlinear odds ratio (OR) in different MLPR levels and integrated it into a logistic model to predict the risk of AKI.

**Results:**

The incidence of hospital-acquired AKI was 18.8% in the derivation cohort. Compared to non-AKI, the MLPR levels were significantly higher in AKI patients. Dose–response curve revealed that the increase of AKI risk was faster in the first half of MLPR and then tended to flatten. After classifying the MLPR levels into six groups, the AKI incidence increased from 4.5% to 55.3% with a peaking OR of 24.38. The AUC values of the AKI model only including MLPR were 0.740, and after gradually integrating other covariates, the area under the receiver operating characteristic (AUC) value reached 0.866, which was significantly higher than the AUC of full models without MLPR (0.822). Moreover, the better prediction ability of AKI was observed in the external validation, with an AUC of 0.899.

**Conclusion:**

MLPR has good predictive efficiency in AKI, which can be used as a simple and easy clinical composite index to effectively predict early pulmonary infection-related AKI.

**Supplementary Information:**

The online version contains supplementary material available at 10.1186/s40001-022-00906-6.

## Introduction

Acute kidney injury (AKI) is one of the most common and serious complications of sepsis, and pulmonary infection is one of its most common etiologies. Studies have shown that approximately 20% of patients with pulmonary infections occur AKI [1, 2]. Recent reports showed that the incidence of AKI can be as high as 40.9% in patients with severe pneumonia caused by COVID-19 in intensive care units [3]. Other studies showed that the incidence of pulmonary infection-related AKI is approximately 30% [1, 2, 4, 5]. The rates of death and adverse renal events after discharge among hospital-acquired pneumonia-associated AKI patients were significantly higher than those with pneumonia or AKI alone [6]. Therefore, it is crucial to identify reliable markers and construct a simple and effective AKI risk prediction model for pulmonary infection-related AKI.

In recent years, peripheral blood leukocyte counts have attracted attention because the immune-inflammatory status is closely related to the occurrence and development of AKI. Previously published articles have also discussed the role of NLR as a predictive factor for AKI in different populations [7–10]. In addition, monocyte macrophages also serve as important innate immune cells that regulate the immune response, and their pathophysiological role is important throughout the process of AKI. It is hypothesized that monocyte-related indicators are reliable biomarkers for AKI prediction. In the current study, we aimed to investigate the predictive value of peripheral monocytes and their related inflammatory indicators, especially monocyte/(lymphocyte × platelet) ratio (MLPR), in pulmonary infection-related AKI, compare the prediction efficacy of MLPR and establish a more convenient and reliable prediction model for early diagnosis pulmonary infection-related AKI.

## Methods

### Study design and patient selection

This was a retrospective study in a general hospital in East China. A total of 2353 patients who were diagnosed with pulmonary infection from January 1 to December 31 2019 were included. The inclusion criteria were as follows: age > 18 years, length of stay ≥ 48 h, and pneumonia diagnosed during hospitalization. Patients were excluded if they were < 18 years old, had already been admitted with AKI, underwent maintenance hemodialysis or renal transplantation, hospital stay of < 24 h, lack of serum creatinine (SCr) test or other biochemical tests, and were without two or more SCr test during hospitalization. After reviewing the medical records, 1038 patients were selected as potentially eligible participants. We further assigned patients admitted from January 1st to November 30th as the derivation cohort, and patients admitted from December 1st to 31st as the validation cohort. This study was approved by the institutional committee of our hospital (B2018-175).

### Pulmonary infection and AKI definition

AKI was defined according to the 2012 KDIGO guideline as any of the following: an increase in SCr ≥ 0.3 mg/dL (≥ 26.5 μmol/L) within 48 h, an increase in SCr to ≥ 1.5 times the baseline that was known or presumed to have occurred within the prior seven days. Severe AKI was defined as KDIGO stage 2 or 3. We defined SCr within 24 h of admission as the baseline creatinine.

Pulmonary infection was diagnosed according to the 2018 pneumonia guidelines, with typical clinical symptoms and signs as follows: temperature > 38.0 °C; recent onset of cough, sputum, or dyspnea; peripheral white blood cell count < 4 × 10^9^ /L or > 10 × 10^9^ /L; dry and wet rales or sputum rales heard from lung auscultation; and meet one of the following conditions: chest X-ray examination or CT had lung invasive inflammation or culture-positive sputum or tracheobronchial secretion [6].

### Data collection

Demographic characteristics, including age, gender, height, weight, body mass index (BMI), and comorbidities (hypertension, diabetes mellitus, coronary heart disease, stroke, chronic kidney disease, and malignancy), were collected according to the electronic medical record system.

The biochemical data of the patients within 24 h of admission were collected as baseline variables, including renal function: blood urea nitrogen (BUN), SCr, eGFR, and uric acid; liver function: aspartate transaminase (AST), alanine aminotransferase (ALT), and total bilirubin (TBIL); electrolyte: blood sodium, potassium, chloride, calcium, magnesium, phosphorus, and CO_2_; blood gas analysis: pH, partial pressure of CO_2_, O_2_, HCO_3_^−^, and base excess; and other biochemical data: hemoglobin, hematocrit, platelet count, albumin, and globulin; inflammatory indicators: procalcitonin(PCT),C-reactive protein (CRP) and sputum culture data.

Peripheral blood indexes were collected as the time of the diagnosis of pulmonary infection, including leukocyte, neutrophil, lymphocyte, and monocyte counts. The composite inflammatory indexes of MLPR and NLPR were calculated as follows:

$$\mathrm{MLPR}=\frac{\mathrm{Monocyte\, count }({10}^{9}/\mathrm{L}) \times 1000}{\mathrm{Lymphocyte \,count }({10}^{9}/\mathrm{L})\times \mathrm{platelets }({10}^{9}/\mathrm{L})},$$

$$\mathrm{NLPR}=\frac{\mathrm{Neutrophil\, count }({10}^{9}/\mathrm{L}) \times 100}{\mathrm{Lymphocyte\, count }({10}^{9}/\mathrm{L})\times \mathrm{platelets }({10}^{9}/\mathrm{L})}.$$

### Statistical analysis

The statistical analysis was run in R 3.6.1 software (R core team). The data of normal distribution are described as mean ± standard deviation, and the data of skewed distribution are described by the median and interquartile range and were compared by Student’s t-test and Wilcoxon test. Categorical variables were described by frequency (Hmisc package) and compared using Pearson’s test ("gmodmodelsckage). For dose–response relationship statistics, a correlation model between peripheral blood leukocyte counts composite inflammatory indexes, and the incidence of AKI related to pulmonary infection, was constructed at 10%, 50%, and 90%, and the likelihood ratio test was used to compare nonlinear variables (‘‘tidyverse’’, ‘‘rms’’, and ‘‘ggplot2’’ packages). In addition, we used logistic regression to analyze the multivariable, including composite inflammatory index, and gradually added parameters to create six models: Model 1, which only included the baseline composite inflammatory index to the full model; Model 2, based on Model 1, including basic demographic data (age, sex, and BMI) and hypertension, diabetes, coronary heart disease, stroke, chronic kidney disease, and malignant tumor; Model 3, based on Model 2, including baseline liver and kidney functions (AST, ALT, eGFR, and uric acid) and blood index(albumin and hematocrit); Model 4, based on Model 3, including inpatient diagnosis and treatment (emergency admission, surgery, and nephrotoxic drugs); Model 5, based on Model 4, removing only the baseline composite inflammatory index; Model 6, full model by using validation dataset. The predictive ability of baseline composite inflammatory indexes was quantitatively evaluated by the area under the receiver operating characteristic curve (AUC) (‘‘pROC’’ ‘‘and ggplot2’’ packages). Significance comparisons of the AUC value difference between models were performed using the Delong test. All statistical tests were two-tailed, and the statistical significance was set at *p* < 0.05.

## Results

### Baseline characteristics and AKI incidence

**Fig. 1:**
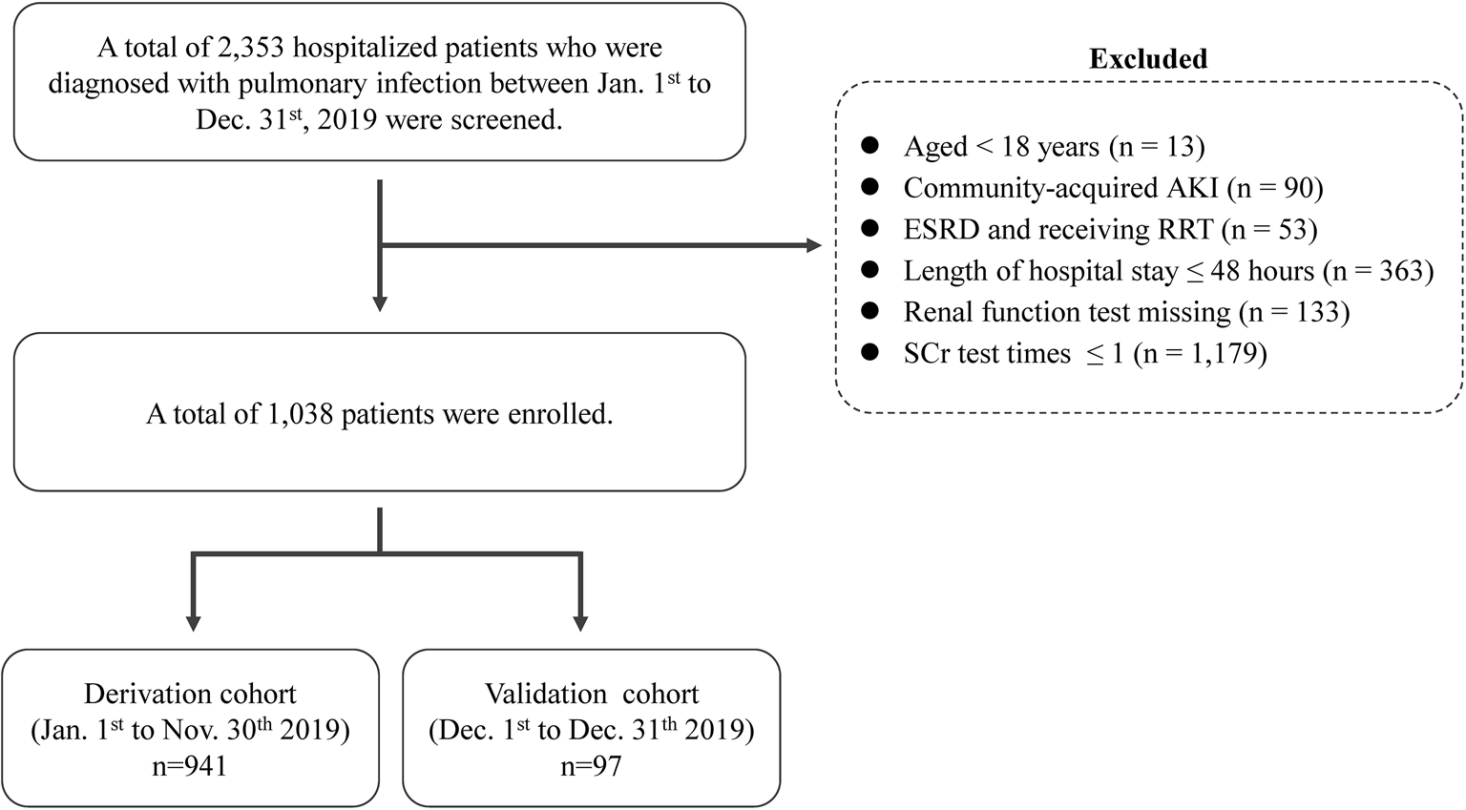
Flowchart of the selection process of eligible studies. ESRD: end stage renal disease; SCr: serum creatinine

Of the 2353 hospitalized patients who were diagnosed with pulmonary infection, 1038 met the inclusion criteria and were then divided into the derivation cohort (*n* = 941) and the validation cohort (*n* = 97, Fig. 1). Additional file 1: Table S1 suggests that most of the variables’ statistical distributions between the derivation and validation cohorts were comparable. Additional file 3: Fig. S1 shows the pathogen and etiologies of pneumonia. The main pathogen included mycobacterium, *Acinetobacter baumannii, Klebsiella pneumoniae*, *Pseudomonas aeruginosa*.

In the derivation cohort, the mean age was 65.31 ± 16.55 years, and 633 (67.3%) were male. According to the KDIGO classification, 177 (18.8%) patients developed AKI during hospitalization. Among them, 92 (9.8%), 44 (4.7%) and 41 (4.4%) patients were in AKI stage 1, stage 2 and stage 3, respectively. The proportion of patients with AKI who received renal replacement therapy was 5.6% (10/177). Table 1 shows the major factors associated with pulmonary infection-related AKI. Patients who were older and male were more likely to develop AKI. Previous comorbidities of coronary heart disease and CKD were positively associated with AKI (aOR = 1.66 and 6.30 for AKI). Moreover, the risk of AKI was significantly increased in patients who underwent emergency hospitalization, surgery, or nephrotoxic drug treatment, with the aOR of 1.63, 1.63, and 2.79, respectively.

**Table 1:** Demographic characteristics

Variables	AKI (*n* = 177)	non-AKI (*n* = 764)	Total (*n* = 941)	AKI		Severe AKI	
				OR (95%CI)	*P*	OR (95%CI)	*P*
Demographic characteristics							
Age	73.67 ± 15.19	63.37 ± 16.25	65.31 ± 16.55	1.05 (1.03–1.06)	< 0.001	1.04 (1.03–1.06)	< 0.001
Male gender, *n* (%)	133 (75.1)	500 (65.4)	633 (67.3)	1.59 (1.09–2.35)	0.019	1.50 (0.91–2.58)	0.127
BMI(kg/m^2^)	22.55 ± 3.58	22.09 ± 3.80	22.18 ± 3.76	1.04 (0.99–1.08)	0.130	1.01 (0.95–1.07)	0.773
Comorbidities							
Hypertension, *n* (%)	65 (21.3)	240 (78.7)	305 (100.0)	0.79 (0.54–1.15)	0.222	0.84 (0.50–1.36)	0.480
Diabetes mellitus, *n* (%)	29 (20.7)	111 (79.3)	140 (100.0)	0.96 (0.59–1.51)	0.848	0.72 (0.34–1.37)	0.345
Coronary heart disease, *n* (%)	53 (32.7)	109 (67.3)	162 (100.0)	1.66 (1.10–2.48)	0.015	1.68 (0.97–2.85)	0.060
Stroke, *n* (%)	35 (33.3)	70 (66.7)	105 (100.0)	1.70 (1.05–2.70)	0.027	1.93 (1.03–3.48)	0.033
CKD, *n* (%)	84 (51.5)	79 (48.5)	163 (100.0)	6.30 (4.22–9.45)	< 0.001	5.04 (2.97–8.52)	< 0.001
Malignancy, *n* (%)	43 (18.4)	191 (81.6)	234 (100.0)	1.06 (0.70–1.57)	0.787	1.20 (0.69–2.02)	0.505
Treatment							
Emergency, *n* (%)	94 (24.9)	283 (75.1)	377 (100.0)	1.63 (1.16–2.31)	0.005	1.67 (1.05–2.67)	0.030
Surgery, *n* (%)	31 (25.0)	93 (75.0)	124 (100.0)	1.63 (1.01–2.57)	0.041	1.44 (0.72–2.69)	0.274
Nephrotoxic drug, *n* (%)	136 (24.5)	418 (75.5)	554 (100.0)	2.79 (1.90–4.17)	< 0.001	2.50 (1.50–4.29)	< 0.001

OR values for age, gender, and BMI were calculated in univariate model, and the rest OR values of comorbidities and treatment were adjusted for age, sex and BMI

*BMI* body mass index, *CKD* chronic kidney disease

### Association between AKI and clinical laboratory biochemical data

The risk of pulmonary infection-related AKI was significantly increased in patients with high BUN, SCr, uric acid, AST, TBIL and PCT (within 24 h of admission). These associations remained significant after adjusting for demographic factors (Table 2). Hemoglobin, hematocrit, and albumin levels negatively correlated with pulmonary infection-related AKI. Notably, the platelet count in AKI patients was significantly lower than that in non-AKI patients (137.36 ± 88.07 vs. 208.55 ± 101.54, aOR = 0.92, 95% CI 0.90–0.94). PCT in AKI patients was significantly higher than that in non-AKI patients (0.40 vs.0.10, aOR = 1.41, 95% CI 1.23–1.63). Further analysis of severe AKI revealed a similar pattern.

**Table 2:** Clinical biochemical indexes and pulmonary infection-related AKI

Variables	AKI (*n* = 177)	non-AKI (*n* = 764)	Total (*n* = 941)	AKI		Severe AKI	
				aOR (95% CI)	*P*	aOR (95% CI)	*P*
Renal function							
BUN (mmol/L)	9.78 ± 6.40	5.85 ± 3.11	6.59 ± 4.23	1.23 (1.17–1.29)	< 0.001	1.20 (1.14–1.28)	< 0.001
SCr (µmol/L)	111.55 ± 62.95	72.82 ± 29.35	80.11 ± 40.87	1.02 (1.02–1.03)	< 0.001	1.02 (1.01–1.02)	< 0.001
eGFR (ml/min/1.73m^2^)	64.14 ± 27.79	90.11 ± 23.89	85.23 ± 26.67	0.96 (0.96–0.97)	< 0.001	0.97 (0.96–0.98)	< 0.001
Uric acid (µmol/L)^a^	362.29 ± 165.90	266.96 ± 117.80	284.89 ± 133.44	1.05 (1.04–1.06)	< 0.001	1.04 (1.02–1.06)	< 0.001
Liver function							
AST (IU/L)^a^	26 [17–43]	23 [16–33]	23 [16–34]	1.05 (1.02–1.08)	< 0.001	1.05 (1.02–1.08)	0.005
ALT (IU/L)^a^	19 [12–37]	21 [13–36]	21 [13–36]	1.02 (1.00–1.05)	0.194	1.02 (1.00–1.05)	0.146
Total bilirubin (µmol/L)	14.93 ± 15.47	11.94 ± 13.59	12.51 ± 14.00	1.02 (1.00–1.03)	0.006	1.02(1.00–1.03)	0.006
Other laboratory indexes							
Hemoglobin (g/L)^a^	111.45 ± 25.51	116.68 ± 23.26	115.70 ± 23.38	0.89 (0.82–0.96)	0.003	0.91 (0.82–1.01)	0.074
Hematocrit (L/L)	33.61 ± 7.19	35.10 ± 6.68	34.82 ± 6.80	0.96 (0.94–0.99)	0.003	0.97 (0.93–1.00)	0.050
Platelet (10^9^/L)^a^	137.36 ± 88.07	208.55 ± 101.54	195.16 ± 102.94	0.92 (0.90–0.94)	< 0.001	0.85 (0.82–0.89)	< 0.001
Total protein (g/L)	63.12 ± 8.36	64.79 ± 8.82	64.47 ± 8.76	0.98 (0.96–1.00)	0.060	0.99 (0.96–1.02)	0.374
Albumin (g/L)	34.65 ± 5.37	36.01 ± 6.28	35.76 ± 6.14	0.96 (0.94–0.99)	0.020	0.96 (0.92–1.00)	0.050
Globulin (g/L)	28.44 ± 7.21	28.78 ± 6.45	28.72 ± 6.60	1.00 (0.97–1.03)	0.949	1.01 (0.97–1.04)	0.599
Albumin and globulin ratio	1.29 ± 0.36	1.31 ± 0.37	1.31 ± 0.37	0.79 (0.49–1.28)	0.345	0.77 (0.39–1.46)	0.427
CRP (mg/L)	46.3 [15.6–125.5]	36.9 [10.3–94.1]	38.3 [11.2–96.6]	1.00 (1.00–1.00)	0.271	1.00 (1.00–1.01)	0.017
PCT (ng/mL)	0.40 [0.15–1.17]	0.10[0.05–0.32]	0.13 [0.06–0.44]	1.41 (1.23–1.63)	< 0.001	1.50 (1.27–1.75)	< 0.001
Electrolyte							
Sodium (mmol/L)^a^	139.67 ± 6.05	138.89 ± 4.54	139.04 ± 4.87	1.22 (0.87–1.72)	0.241	0.94 (0.58–1.52)	0.789
Potassium (mmol/L)	4.02 ± 0.57	3.91 ± 0.59	3.93 ± 0.59	1.41 (1.04–1.95)	0.031	1.31 (0.87–2.01)	0.215
Chlorine (mmol/L)^a^	102.68 ± 6.51	101.39 ± 4.98	101.64 ± 5.32	1.45 (1.06–2.00)	0.020	1.21 (0.77–1.90)	0.416
Calcium (mmol/L)	2.14 ± 0.20	2.16 ± 0.17	2.16 ± 0.18	0.57 (0.19–1.72)	0.321	0.86 (0.20–3.59)	0.834
Phosphorus (mmol/L)	1.01 ± 0.34	1.00 ± 0.27	1.01 ± 0.28	1.92 (0.92–3.99)	0.082	1.17 (0.45–3.07)	0.750
Magnesium (mmol/L)	0.85 ± 0.12	0.86 ± 0.10	0.85 ± 0.10	0.47 (0.07–3.03)	0.427	0.17 (0.01–1.99)	0.159
CO_2_ (mmol/L)	25.26 ± 4.07	26.08 ± 3.52	25.92 ± 3.65	0.91 (0.87–0.96)	< 0.001	0.89 (0.84–0.95)	< 0.001
Arterial blood gas analysis							
pH	7.42 ± 0.09	7.44 ± 0.32	7.43 ± 0.29	1.00 (0.94–1.09)	0.926	1.01 (0.95–1.13)	0.846
PCO_2_ (kPa)	40.40 ± 13.27	39.41 ± 9.38	39.60 ± 10.24	1.00 (0.98–1.02)	0.771	0.98 (0.95–1.01)	0.209
PO_2_ (kPa)	92.16 ± 45.84	85.23 ± 32.56	86.55 ± 35.53	1.01 (1.00–1.01)	0.051	1.01 (1.00–1.01)	0.050
HCO_3_^−^ (mmol/L)	25.51 ± 6.29	27.15 ± 4.89	26.83 ± 5.23	0.93 (0.89–0.97)	< 0.001	0.91 (0.86–0.96)	0.002
Base excess (mmol/L)	0.3 [-2.8–4.3]	2.6 [0.3–5.2]	2.5 [0.0–5.2]	0.91 (0.87–0.94)	< 0.001	0.86 (0.81–0.92)	< 0.001

*AKI* acute kidney injury, *BUN* blood urea nitrogen, *SCr* serum creatinine, *eGFR* estimated glomerular filtration rate, *AST* aspartate transaminase, *ALT* alanine transaminase, *CO*_*2*_ Carbon dioxide, *PCO*_*2*_ carbon dioxide partial pressure, *PO*_*2*_: oxygen partial pressure, *HCO*_*3*_^*−*^ bicarbonate ion aOR was adjusted by age, gender, and body mass index, *CRP* C-reactive protein, *PCT* procalcitonin

^a^OR increased by 10 units

^b^OR increased by 0.1 units

### Peripheral white cell count and pulmonary infection-related AKI

Peripheral white cell analysis results are summarized in Table 3. Neutrophil counts in the AKI patients were 12.28 ± 6.95 compared to 8.50 ± 5.86 in the non-AKI patients (aOR = 1.09 (95% CI 1.07–1.12)). Similarly, monocyte counts were 0.98 ± 0.50 and 0.84 ± 0.51 in the AKI and non-AKI patients (aOR = 1.66, 95% CI 1.21–2.33). Lymphocyte counts were negatively associated with pulmonary infection-related AKI risk (0.65 ± 0.49 vs. 1.02 ± 0.85, aOR = 0.24, 95% CI 0.16–0.36). For the calculated composite inflammatory indexes, MLPR and NLPR levels were significantly higher in AKI patients (13.14 [5.63–58.75] vs. 4.26 [2.19–8.71], *p* < 0.001; 15.98 [4.84–81.23] vs. 3.87 [2.04–9.06], *p* < 0.001). Multivariate analysis showed that the risk of AKI increased when MLPR and NLPR were elevated, with aORs of 1.01 (95% CI 1.01–1.02) and 1.01 (95% CI 1.01–1.01), respectively. In the analysis of severe AKI, the ORs also increased with MLPR and NLPR expression.

**Table 3:** Multivariate logistic regression analysis of peripheral leukocyte count, composite inflammatory indexes

Variables	AKI (*n* = 177)	non-AKI (*n* = 764)	Total (*n* = 941)	AKI		Severe AKI	
				aOR (95% CI)	*P*	aOR (95% CI)	*P*
Peripheral blood leukocytes count							
Leukocyte count (10^9^/L)	14.30 ± 7.05	10.82 ± 6.58	11.48 ± 6.81	1.08 (1.05–1.10)	< 0.001	1.12 (1.08–1.15)	< 0.001
Neutrophil count (10^9^/L)	12.28 ± 6.95	8.50 ± 5.86	9.21 ± 6.25	1.09 (1.07–1.12)	< 0.001	1.14 (1.11–1.18)	< 0.001
Monocyte count (10^9^/L)	0.98 ± 0.50	0.84 ± 0.51	0.87 ± 0.51	1.66 (1.21–2.33)	< 0.001	2.04 (1.36–3.17)	< 0.001
Lymphocyte count (10^9^/L)	0.65 ± 0.49	1.02 ± 0.85	0.95 ± 0.81	0.24 (0.16–0.36)	< 0.001	0.05 (0.02–0.10)	< 0.001
Composite inflammatory indexes							
MLPR	13.14 [5.63–58.75]	4.26 [2.19–8.71]	4.91 [2.39–11.72]	1.01 (1.01–1.02)	< 0.001	1.01 (1.01–1.02)	< 0.001
NLPR	15.98 [4.84–81.23]	3.87 [2.04–9.06]	4.56 [2.22–13.39]	1.01 (1.01–1.01)	< 0.001	1.01 (1.01–1.01)	< 0.001

*MLPR* monocytes/(lymphocytes × platelet) ratio, *NLPR* neutrophils/(lymphocytes × platelet) ratio, *aOR* was adjusted by age, gender, and body mass index

### Dose–response relationship of composite inflammatory indexes and pulmonary infection-related AKI

**Fig. 2:**
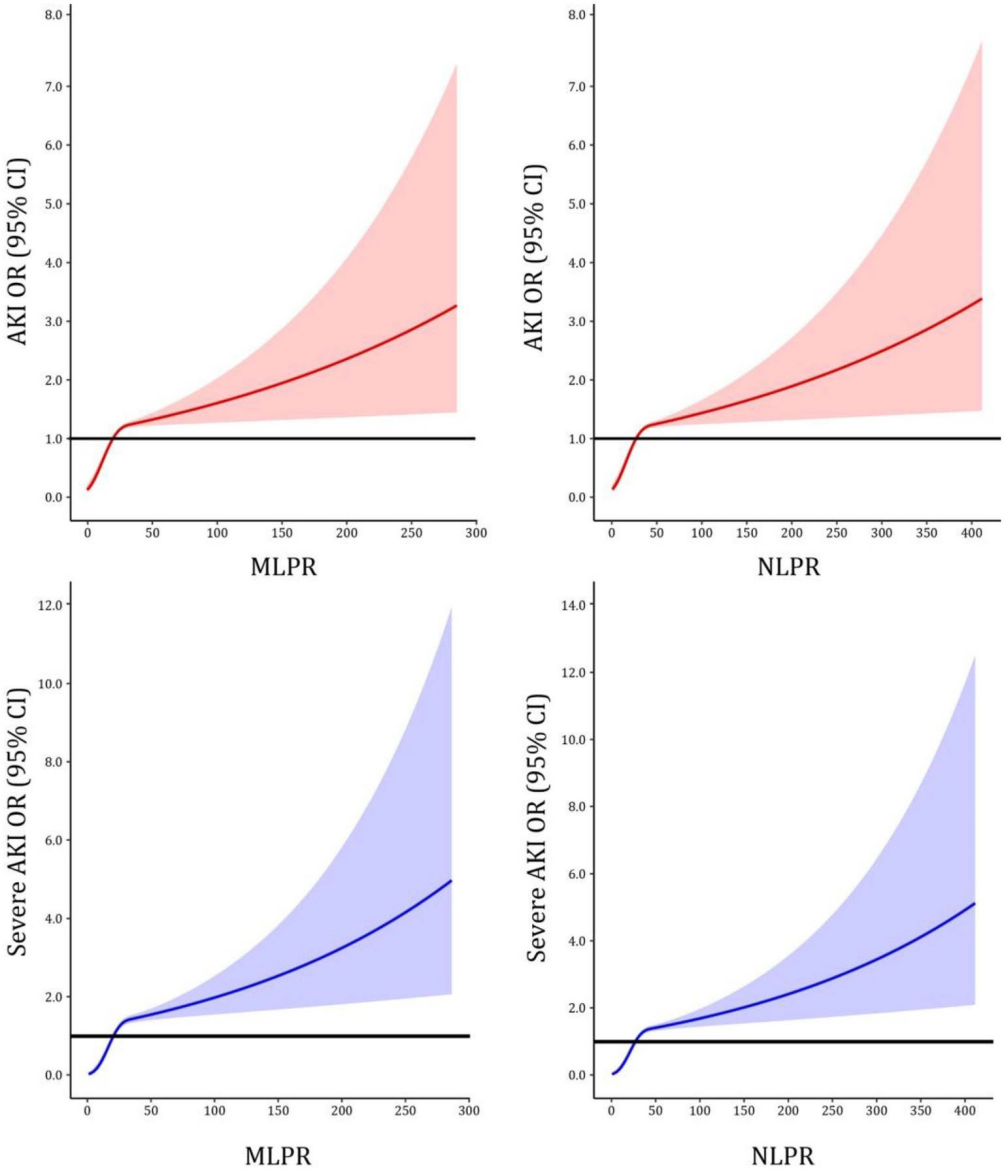
Dose–response relationship of peripheral white blood cell counts, composite inflammatory biomarkers, and pulmonary infection-associated AKI and severe AKI

As shown in Fig. 2, we used restricted cubic splines to visualize the dose–response relationship between composite inflammatory indexes and pulmonary infection-related AKI. MLPR and NLPR were positively correlated with AKI occurrence, and the upward trends could be divided into two parts. The increase in AKI risk at MLPR < 40 (aOR_MLPR<40_ = 1.07 (95% CI: 1.05–1.09)) was faster than that at MLPR ≥ 40 (aOR_MLPR≥40_ = 1.00 (95% CI: 1.00–1.01)). Similarly, the rate of increase in the risk of AKI with NLPR < 48 (aOR_NLPR<48_ = 1.05 (95% CI: 1.03–1.07)) was faster than that with NLPR ≥ 48 (aOR_NLPR≥48_ = 1.00 (95% CI: 1.00–1.01)). We then classified the MLPR and NLPR values into six-level grades (Table 4). Compared with the MLPR level of 0–1.9, the risk continued to grow at higher MLPR levels (incidence: 4.5% to 55.3%, aOR: 2.02 to 24.38). Regarding NLPR, the incidence of AKI increased remarkably from 3.6% in the reference level to 51.1% at the highest level. Similar upward trends were found in severe AKI but with greater aOR estimates. After dividing the CRP and PCT into five grades, we found that PCT group showed a linear increasing trend in AKI and severe AKI (Additional file 2: Table S2).

**Table 4:** Levels of NLPR and MLPR and risk stratification for AKI

	*n*	AKI			Severe AKI		
		*n* (%)	aOR (95% CI)	P	*n* (%)	aOR (95% CI)	*P*
MLPR							
0–1.9	177	8 (4.5)	Ref.	–	1 (0.6)	Ref.	–
2.0–3.9	218	24 (11.0)	2.02 (0.90–5.01)	0.105	4 (1.8)	2.56 (0.37–50.83)	0.406
4.0–7.9	220	31 (14.1)	2.27 (1.03–5.52)	0.050	9 (4.1)	5.20 (0.94–97.17)	0.123
8.0–15.9	146	34 (23.3)	4.91 (2.23–12.01)	< 0.001	15 (10.3)	18.75 (3.62–344.76)	0.005
16.0–31.9	77	23 (29.9)	6.11 (2.56–15.92)	< 0.001	10 (13.0)	22.59 (3.95–428.02)	0.004
≥ 32.0	103	57 (55.3)	24.38 (11.10–60.10)	< 0.001	46 (44.7)	161.40 (32.72–2931.98)	< 0.001
NLPR							
0–1.9	196	7 (3.6)	Ref.	–	1 (0.5)	Ref.	–
2.0–3.9	233	29 (12.4)	3.02 (1.34–7.79)	0.012	6 (2.6)	4.09 (0.67–78.51)	0.198
4.0–7.9	182	30 (16.5)	3.83 (1.68–9.88)	0.003	9 (4.9)	8.41 (1.50–157.74)	0.047
8.0–15.9	126	23 (18.3)	5.18 (2.20–13.69)	< 0.001	9 (7.1)	14.74 (2.63–277.24)	0.012
16.0–31.9	73	21 (28.8)	8.35 (3.38–22.89)	< 0.001	6 (8.2)	17.81 (2.81–347.38)	0.010
≥ 32.0	131	67 (51.1)	26.59 (12.01–67.90)	< 0.001	54 (41.2)	160.06 (32.64–2906.32)	< 0.001

### Prediction model of AKI based on MLPR and NLPR

**Fig. 3:**
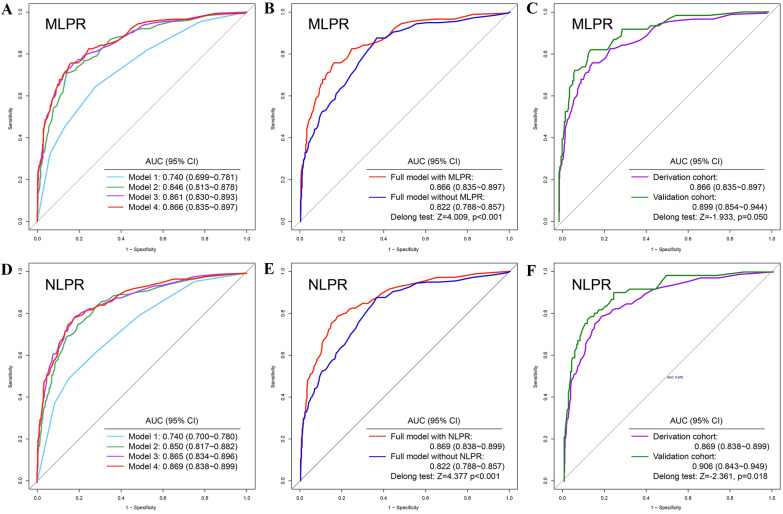
The efficacy of prediction models for AKI with different composite inflammatory biomarkers. **A** In the model with MLPR alone, the AUC value was 0.740. With the progressive enrollment of variables, the AUC values increased from 0.846, 0.861 to 0.866. **B** The Delong test revealed that the AUC of the full model with MLPR was significantly higher than that of the full model without MLPR (AUC: 0.866 vs. 0.822, *Z* = 4.009, *P* < 0.001). **C** In validation cohort, the AUC of full model reached 0.899. **D** In the model with NLPR alone, the AUC value was 0.740. With the progressive enrollment of variables, the AUC values increased from 0.850, 0.865 to 0.869. **E** The Delong test revealed that the AUC of the full model with NLPR was significantly higher than that of the full model without NLPR (AUC: 0.869 vs. 0.822, *Z* = 4.377, *P* < 0.001). **F** In validation cohort, the AUC of full model reached 0.906

**Fig. 4:**
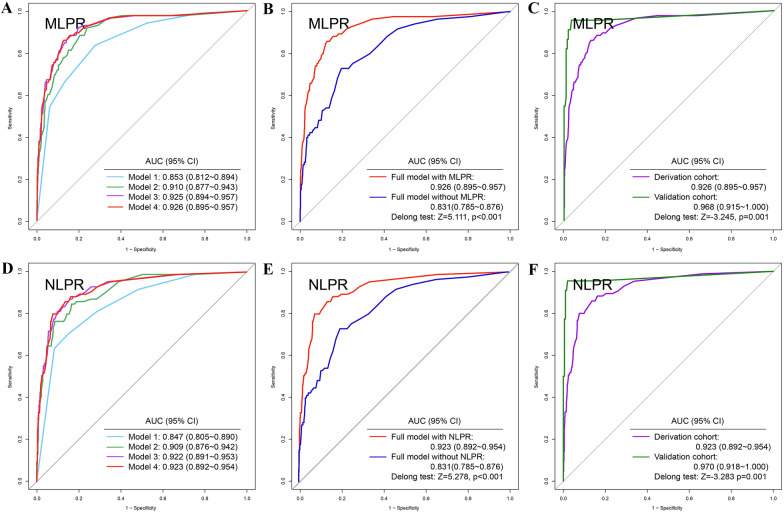
The efficacy of prediction models for severe AKI with different composite inflammatory biomarkers. **A** In the model with MLPR alone, the AUC value was 0.853. With the progressive enrollment of variables, the AUC values increased from 0.910, 0.925 to 0.926. **B** The Delong test revealed that the AUC of the full model with MLPR was significantly higher than that of the full model without MLPR (AUC: 0.926 vs. 0.831, *Z* = 5.111, *p *< 0.001). **C** In validation cohort, the AUC of full model reached 0.968. **D** In the model with NLPR alone, the AUC value was 0.847. With the progressive enrollment of variables, the AUC values increased from 0.909, 0.922 to 0.923. **E** The Delong test revealed that the AUC of the full model with NLPR was significantly higher than that of the full model without NLPR (AUC: 0.923 vs. 0.831, *Z* = 5.278, *P* < 0.001). **F** In validation cohort, the AUC of full model reached 0.970

We used a logistic model based on composite inflammatory indexes to predict AKI (Fig. 3). In the model with MLPR alone, the AUC value was 0.740 (95% CI: 0.699–0.781). With the progressive enrollment of variables, the AUC values increased from 0.846 (95% CI: 0.813–0.878) to 0.861 (95% CI: 0.830–0.893) and then to 0.866 (95% CI: 0.835–0.897). The AUC of the MLPR model was comparable to that of the NLPR model. We further evaluated the attribution of the MLPR to AKI in the full model. The Delong test revealed that the AUC of the model with MLPR was significantly higher than that of the model without MLPR (AUC: 0.866 vs. 0.822, Z = 4.009, *p* < 0.001). In external validation, the AUC of the full model reached 0.899 (95% CI: 0.854–0.944), suggesting a better prediction ability (*p* = 0.050). In the analysis of severe AKI, the predictive capacities of the MLPR in internal and external full models were 0.926 (95% CI: 0.895–0.957) and 0.968 (95% CI: 0.915–1.000), respectively (Fig. 4). In the model with CRP or PCT, the AUC value for AKI were 0.547, 0.729, respectively. It was found that the AUC values of NLPR and MLPR were higher than those included CRP and PCT Additional file 4: Fig. S2).

## Discussion

Our study demonstrated a significant correlation between pulmonary infection-associated AKI and the composite inflammatory index of MLPR. A comprehensive comparison of AKI prediction models showed that MLPR achieved good predictive performance for AKI and severe AKI, suggesting that MLPR can be used as a predictor of AKI and severe AKI.

Composite inflammatory indicators are easily obtained, calculated, and low-cost. Previous studies have suggested that immune-inflammatory cells play an important role in the occurrence and development of kidney diseases [11–14]. For instance, monocytes and macrophages are closely related to AKI [13]. When the kidney is injured, peripheral blood monocytes are recruited to the kidney chemotactically and further differentiate into macrophages. Macrophages are heterogeneous and plastic [15]. M1 macrophages have pro-inflammatory effects, whereas M2 macrophages have anti-inflammatory and pro-reparative effects. The results of this study showed that MLPR could achieve good predictive performance for AKI and severe AKI, the AUC of which was comparable to NLPR. This may be due to the role of macrophages in AKI development. Damage-related molecular patterns and pathogen-associated molecular patterns (such as lipopolysaccharide) can quickly induce the recruitment of a huge number of neutrophils and monocytes to the kidney. Monocytes pass through the endothelium, then migrate to kidney tissue, and differentiate into macrophages. Based on the opposite function of M1 and M2 macrophages, an increasing number of studies have focused on inhibiting the activation of M1 macrophages and promoting the activation of M2 macrophages to reduce tubular damage, enhance repair, inhibit inflammation, activate collagen remodeling, and prevent kidney fibrosis in the last stage [16]. Yang et al. demonstrated complementary roles of kidney-resident macrophages and monocyte-derived infiltrating macrophages in modulating tissue inflammation and promoting tissue repair. These findings support the S100a8/a9 + blockade as a feasible and clinically therapeutic potential for human AKI [17]. However, the current data on macrophage activation in ischemic kidney injury are derived from animal models. This has not been confirmed in humans, because renal biopsies are rarely performed in patients with acute tubular necrosis. Once confirmed, the mechanisms that control macrophage activation and effects may enable targeted therapy for ischemic kidney injury in the future [18].

Our study found that MLPR can achieve a good predictive performance, which may be due to the involvement of both lymphocytes and platelets in the pathogenesis of AKI. The results of this study showed that there was a negative "linear" relationship between lymphocyte, platelet counts, and the risk of AKI. Low lymphocyte levels are common in moderate to severe bacterial infections, and significantly elevated lymphocyte levels are more common in viral infections. T helper 1 and 2 cells have been shown to contribute to the pathophysiology of AKI in both animals and humans [19]. In the pathophysiological process of AKI, the fibrinolytic system is disordered and always accompanied by a hemorrhagic tendency, which is closely related to platelet function. Platelets participate in various inflammatory processes and the pathogenesis of AKI and are recruited to injured tissues in the early stages of inflammation and adhesion to the inner wall of blood vessels. Subsequently, platelets exacerbate local or systemic inflammatory responses by inducing neutrophil infiltration through the release of inflammatory substances [20–22]. Therefore, MLPR may be more suitable than MLR for predicting the occurrence of inflammation-associated AKI.

In the present study, MLPR achieved good predictive performance for AKI and severe AKI, with a trend similar to that of NLPR. Previous studies have found that an elevated NLR and NLPR are independently associated with AKI in patients with sepsis and major abdominal surgery [23–26]. Li et al. found that the dynamic increase in perioperative NLPR significantly increased the risk of AKI during the cardiac perioperative period [27]. In patients with severe sepsis, the NLR can better predict the occurrence of AKI than CRP and white blood cells [28]. In our study, we also found that MLPR and NPLR have better predictive values for AKI than PCT and CRP. In addition, blood routine examination is a must-check item for hospitalized patients, MLPR and NLPR have better clinical generalization of predicting AKI than CRP and PCT.

This study had some limitations. First, this study was a single-center study, and the validation cohort was derived from the same source population. Potential selection bias was inevitable. Second, cytokines examination was not performed in each patient, such as interleukin (IL)-6, IL-8, IL-10, and tumor necrosis factor-α (TNF-α). Third, we did not include some potential protective or risk factors for AKI into analysis, for example, drugs or circumstances of blood sugar control, because most medication and the messages of sugar control cannot be grabbed from our medical record. In future studies, multi-center studies should be conducted to validate the predictive value of composite inflammatory indicators in the risk of infection-associated AKI.

In conclusion, MLPR has good predictive efficiency for pulmonary infection-associated AKI and severe AKI. The predictive ability of MLPR is as good as that of NLPR, which suggests that MLPR can also be used as a simple and easy clinical composite index to predict infection-related AKI.

## Supplementary Information

**Additional file 1: Table S1.** Distribution of demographic and clinical factors in the derivation cohort and validation cohorts.

**Additional file 2: Table S2.** Levels of CRP and PCT and risk stratification for AKI.

**Additional file 3: Figure S1.** The pathogen and etiologies of pneumonia

**Additional file 4: Figure S2.** The efficacy of prediction model for AKI and severe AKI with different composite inflammatory biomarkers.

## Data Availability

Some or all data, models, or code generated or used during the study are available in a repository or online in accordance with funder data retention policies.
