# Supplementary material for: The ratio of monocytes to lymphocytes multiplying platelet predicts incidence of pulmonary infection-related acute kidney injury
Source: Eur J Med Res. 2022 Dec 27;27:312. doi: 10.1186/s40001-022-00906-6 (PMC9792935; doi:10.1186/s40001-022-00906-6)
Supplement: Supplementary file 1 — Additional file 1: Table S1. Distribution of demographic and clinical factors in the derivation cohort and validation cohorts. [file 40001_2022_906_MOESM1_ESM.docx]

**Supplemental Table 1. Distribution of demographic and clinical factors in the derivation cohort and validation cohorts.**

| **Variables** | **Derivation cohort**  **(n = 941)** | **Validation cohort**  **(n =97)** | **Statistics** | **P-value** |
| --- | --- | --- | --- | --- |
| **Demographic characteristics** |  |  |  |  |
| Age | 65.31 ±16.54 | 68.99 ± 17.68 | -2.073 | 0.038* |
| Male gender, n (%) | 633 (67.3) | 61 (62.9) | 0.762 | 0.383^†^ |
| BMI（kg/m²） | 22.18 ± 3.76 | 22.64 ± 3.49 | -1.163 | 0.245* |
| **Comorbidities** |  |  |  |  |
| AKI, n (%) | 177 (18.8) | 26 (26.8) | 3.572 | 0.059^†^ |
| Severe AKI, n (%) | 85 (9.0) | 7 (7.2) | 0.359 | 0.549^†^ |
| Hypertension, n (%) | 305 (32.4) | 44 (45.4) | 6.606 | 0.010^†^ |
| Diabetes mellitus, n (%) | 140 (14.9) | 18 (18.6) | 0.922 | 0.337^†^ |
| Coronary heart disease, n (%) | 162 (17.2) | 13 (13.4) | 0.912 | 0.339^†^ |
| Stroke, n (%) | 105 (11.2) | 11 (11.3) | 0.003 | 0.957^†^ |
| CKD, n (%) | 163 (17.3) | 30 (30.9) | 10.755 | 0.001^†^ |
| Malignancy, n (%) | 234 (24.9) | 24 (24.7) | 0.001 | 0.978^†^ |
| **Treatment** |  |  |  |  |
| Emergency, n (%) | 377 (40.1) | 34 (35.1) | 0.924 | 0.337^†^ |
| Surgery, n (%) | 124 (13.2) | 11 (11.3) | 0.262 | 0.609^†^ |
| Nephrotoxic drug, n (%) | 554 (58.9) | 59 (60.8) | 0.138 | 0.710^†^ |
| **Renal function** |  |  |  |  |
| BUN (mmol/L) | 6.59 ± 4.23 | 7.38 ± 3.91 | -1.766 | 0.078* |
| SCr (µmol/L) | 80.11 ± 40.87 | 88.99 ± 45.86 | -2.014 | 0.044* |
| eGFR (ml/min/1.73m²) | 85.23 ± 26.67 | 75.98 ± 26.94 | 3.248 | 0.001* |
| Uric acid (µmol/L) | 284.89 ± 133.44 | 300.21 ± 132.48 | -1.077 | 0.282* |
| **Liver function** |  |  |  |  |
| AST (IU/L) | 23 [16-34] | 21 [16-30] | —— | 0.066^#^ |
| ALT (IU/L) | 21 [13-36] | 17 [12-30] | —— | 0.054^#^ |
| Total bilirubin (µmol/L) | 12.51 ± 14.00 | 10.74 ± 8.07 | 1.215 | 0.225* |
| **Other laboratory indexes** |  |  |  |  |
| Hemoglobin (g/L) | 115.70 ± 23.38 | 114.78 ± 19.82 | 0.372 | 0.710* |
| Hematocrit (L/L) | 34.82 ± 6.80 | 34.96 ± 5.79 | -0.191 | 0.848* |
| Platelet (10⁹/L) | 195.16 ± 102.94 | 179.80 ± 99.73 | 1.403 | 0.161* |
| Total protein (g/L) | 64.47 ± 8.76 | 64.47 ± 10.00 | 0.003 | 0.998* |
| Albumin (g/L) | 35.76 ± 6.14 | 35.51 ± 6.13 | 0.376 | 0.707* |
| Globulin (g/L) | 28.72 ± 6.60 | 28.96 ± 7.99 | -0.330 | 0.741* |
| Albumin and globulin ratio | 1.31 ± 0.37 | 1.31 ± 0.37 | 0.052 | 0.958* |
| CRP (mg/L) | 38.3 [11.2-96.6] | 38.9 [8.6-95.7] | —— | 0.862^#^ |
| PCT (ng/mL) | 0.13 [0.06-0.44] | 0.11 [0.05-0.40] | —— | 0.764^#^ |
| **Electrolyte** |  |  |  |  |
| Sodium (mmol/L) | 139.04 ± 4.87 | 139.71 ± 4.09 | -1.311 | 0.190* |
| Potassium (mmol/L) | 3.93 ± 0.59 | 3.95 ± 0.59 | -0.400 | 0.690* |
| Chlorine (mmol/L) | 101.64 ± 5.32 | 102.48 ± 5.09 | -1.495 | 0.135* |
| Calcium (mmol/L) | 2.16 ± 0.18 | 2.14 ± 0.17 | 0.863 | 0.388* |
| Phosphorus (mmol/L) | 1.01 ± 0.28 | 1.06 ± 0.27 | -1.825 | 0.068* |
| Magnesium (mmol/L) | 0.85 ± 0.10 | 0.85 ± 0.10 | 0.044 | 0.965* |
| CO_2_ (mmol/L) | 25.92 ± 3.65 | 26.11 ± 4.53 | -0.467 | 0.641* |
| **Arterial blood gas analysis** |  |  |  |  |
| pH | 7.43 ± 0.28 | 7.44 ± 0.08 | -0.364 | 0.716* |
| PCO_2_ (kPa) | 39.60 ± 10.24 | 41.70 ± 16.70 | -1.472 | 0.141* |
| PO_2_ (kPa) | 86.54 ± 35.53 | 82.50 ± 25.24 | 0.941 | 0.347* |
| HCO_3_- (mmol/L) | 26.83 ± 5.23 | 28.15 ± 6.98 | -1.890 | 0.059* |
| Base excess (mmol/L) | 2.5 [0.0-5.2] | 3.0 [-0.4-6.9] | —— | 0.317^#^ |
| **Peripheral blood leukocytes count** |  |  |  |  |
| Leukocyte count (10^9^/L) | 11.48 ± 6.81 | 11.99 ± 5.81 | -0.718 | 0.473* |
| Neutrophil count (10^9^/L) | 9.21 ± 6.25 | 9.72 ± 5.50 | -0.769 | 0.442* |
| Monocyte count (10^9^/L) | 0.87 ± 0.51 | 0.95 ± 0.59 | -1.561 | 0.119* |
| Lymphocyte count (10⁹/L) | 0.95 ± 0.81 | 0.96 ± 0.63 | -0.089 | 0.929* |
| **Composite inflammatory indexes** |  |  |  |  |
| MLPR | 4.91 [2.39-11.72] | 5.25 [2.58-12.81] | —— | 0.379^#^ |
| NLPR | 4.56 [2.22-13.39] | 5.38 [2.15-18.86] | —— | 0.318^#^ |

* Student’s t-test; # Mann–Whitney test; † Pearson test;

AKI: acute kidney injury; BMI：body mass index; CKD: chronic kidney disease; BUN: blood urea nitrogen; SCr: serum creatinine; eGFR: estimated glomerular filtration rate; AST: aspartate transaminase; ALT: alanine transaminase; CO2: Carbon dioxide; PCO2: carbon dioxide partial pressure; PO2: oxygen partial; HCO3-: bicarbonate ion; MLPR: monocytes / (lymphocytes × Platelet) ratio (MLPR); NLPR: neutrophils/(lymphocytes × Platelet) ratio; aOR was adjusted by age, gender, and body mass index. CRP：C-reactive protein; PCT: procalcitonin.
