# Supplementary material for: The ratio of monocytes to lymphocytes multiplying platelet predicts incidence of pulmonary infection-related acute kidney injury
Source: Eur J Med Res. 2022 Dec 27;27:312. doi: 10.1186/s40001-022-00906-6 (PMC9792935; doi:10.1186/s40001-022-00906-6)
Supplement: Supplementary file 2 — Additional file 2: Table S2. Levels of CRP and PCT and risk stratification for AKI. [file 40001_2022_906_MOESM2_ESM.docx]

**Additional file 2: Table S2. Levels of CRP and PCT and risk stratification for AKI.**

|  | **n** | **AKI** | | | **Severe AKI** | | |
| --- | --- | --- | --- | --- | --- | --- | --- |
|  |  | **n (%)** | **aOR (95% CI)** | **P** | **n (%)** | **aOR (95% CI)** | **P** |
| **CRP** |  |  |  |  |  |  |  |
| 0-0.05 | 169 | 23 (13.6) | Ref | —— | 7 (4.1) | Ref | —— |
| 0.05-0.09 | 168 | 27 (16.1) | 1.10 (0.60-2.07) | 0.741 | 11 (6.5) | 1.54 (0.58-4.33) | 0.389 |
| 0.09-0.17 | 168 | 26 (15.5) | 1.09 (0.58-2.05) | 0.792 | 10 (6.0) | 1.31 (0.48-3.73) | 0.602 |
| 0.17-0.65 | 168 | 27 (16.1) | 1.13 (0.61-2.12) | 0.691 | 11 (6.5) | 1.49 (0.56-4.20) | 0.429 |
| ≥ 0.65 | 168 | 36 (21.4) | 1.53 (0.85-2.79) | 0.159 | 21 (12.5) | 2.91 (1.23-7.66) | 0.020 |
| **PCT** |  |  |  |  |  |  |  |
| 0-7.5 | 137 | 4 (2.9) | Ref | —— | 2 (1.5) | Ref | —— |
| 7.5-26.8 | 132 | 8 (6.1) | 1.91 (0.58-7.40) | 0.012 | 4 (3.0) | 1.98 (0.67-14.61) | 0.437 |
| 26.8-53.4 | 145 | 20 (13.8) | 4.93 (1.78-17.45) | 0.003 | 4 (2.8) | 2.04 (0.39-14.96) | 0.417 |
| 53.4-123.0 | 137 | 33 (24.1) | 9.24 (3.49-32.04) | < 0.001 | 15 (10.9) | 8.97 (2.43-58.05) | 0.004 |
| ≥ 123.0 | 139 | 45 (32.4) | 14.93 (5.67-51.65) | < 0.001 | 27 (19.4) | 18.25 (5.15-116.49) | <0.001 |

AKI: acute kidney injury; CRP: C-reactive protein; PCT: procalcitonin.
