# Supplementary figures and images for: The ratio of monocytes to lymphocytes multiplying platelet predicts incidence of pulmonary infection-related acute kidney injury
Source: Eur J Med Res. 2022 Dec 27;27:312. doi: 10.1186/s40001-022-00906-6 (PMC9792935; doi:10.1186/s40001-022-00906-6)

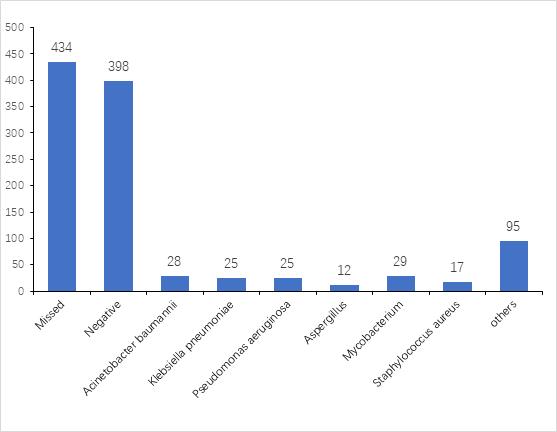

Supplement: Supplementary file 3 — Additional file 3: Figure S1. The pathogen and etiologies of pneumonia [file 40001_2022_906_MOESM3_ESM.jpg]

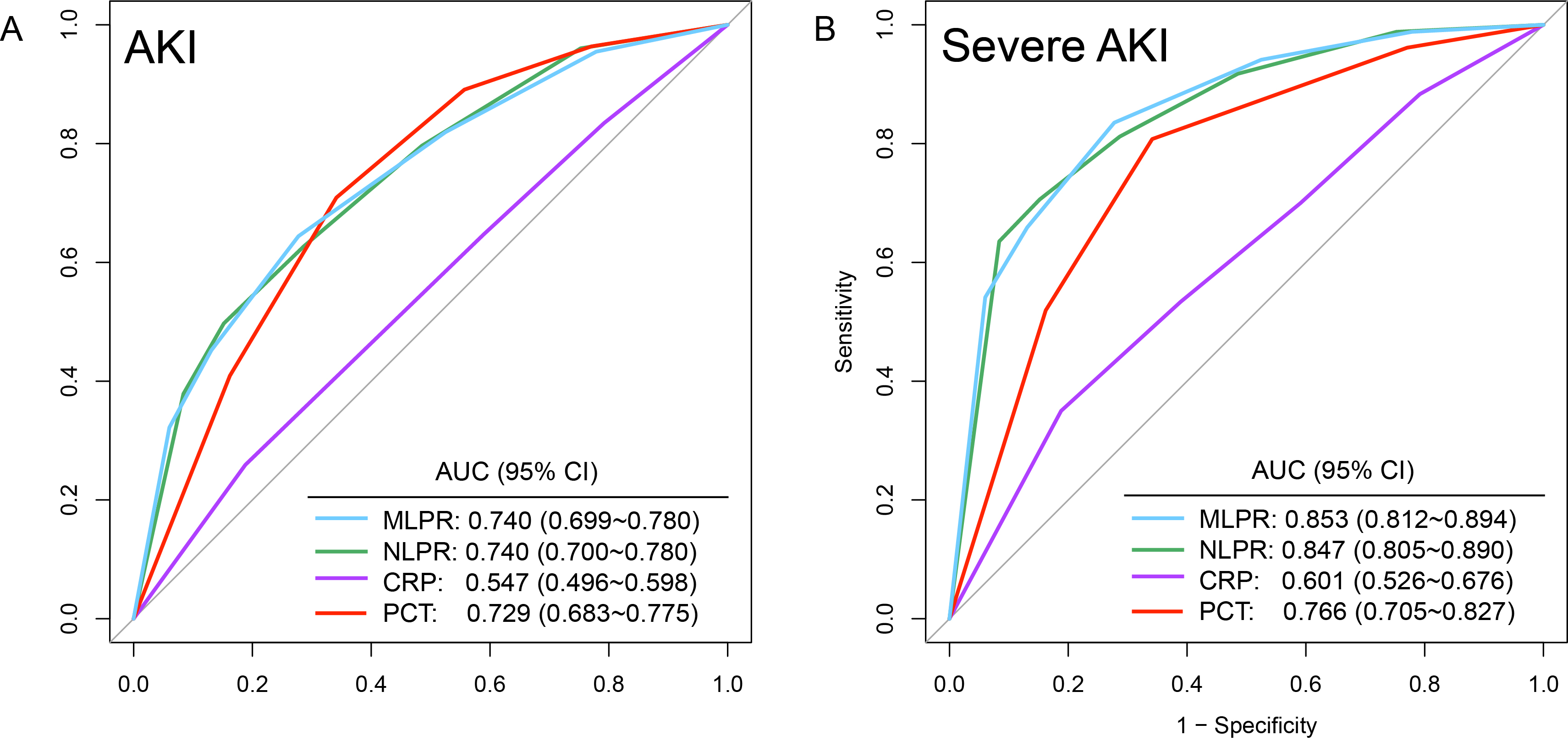

Supplement: Supplementary file 4 — Additional file 4: Figure S2. The efficacy of prediction model for AKI and severe AKI with different composite inflammatory biomarkers. [file 40001_2022_906_MOESM4_ESM.jpg]
